# The Aminopeptidase CD13 Induces Homotypic Aggregation in Neutrophils and Impairs Collagen Invasion

**DOI:** 10.1371/journal.pone.0160108

**Published:** 2016-07-28

**Authors:** Christine A. Fiddler, Helen Parfrey, Andrew S. Cowburn, Ding Luo, Gerard B. Nash, Gillian Murphy, Edwin R. Chilvers

**Affiliations:** 1 Department of Medicine, University of Cambridge School of Clinical Medicine and Addenbrooke’s and Papworth Hospitals, Cambridge, United Kingdom; 2 Papworth Hospital NHS Foundation Trust, Papworth Everard, Cambridge, United Kingdom; 3 Institute of Cardiovascular Sciences, College of Medical and Dental Sciences, University of Birmingham, Birmingham, United Kingdom; 4 Cancer Research UK Cambridge Institute and Department of Oncology, University of Cambridge, Cambridge, United Kingdom; University of Alabama-Birmingham, UNITED STATES

## Abstract

Aminopeptidase N (CD13) is a widely expressed cell surface metallopeptidase involved in the migration of cancer and endothelial cells. Apart from our demonstration that CD13 modulates the efficacy of tumor necrosis factor-α-induced apoptosis in neutrophils, no other function for CD13 has been ascribed in this cell. We hypothesized that CD13 may be involved in neutrophil migration and/or homotypic aggregation. Using purified human blood neutrophils we confirmed the expression of CD13 on neutrophils and its up-regulation by pro-inflammatory agonists. However, using the anti-CD13 monoclonal antibody WM-15 and the aminopeptidase enzymatic inhibitor bestatin we were unable to demonstrate any direct involvement of CD13 in neutrophil polarisation or chemotaxis. In contrast, IL-8-mediated neutrophil migration in type I collagen gels was significantly impaired by the anti-CD13 monoclonal antibodies WM-15 and MY7. Notably, these antibodies also induced significant homotypic aggregation of neutrophils, which was dependent on CD13 cross-linking and was attenuated by phosphoinositide 3-kinase and extracellular signal-related kinase 1/2 inhibition. Live imaging demonstrated that in WM-15-treated neutrophils, where homotypic aggregation was evident, the number of cells entering IL-8 impregnated collagen I gels was significantly reduced. These data reveal a novel role for CD13 in inducing homotypic aggregation in neutrophils, which results in a transmigration deficiency; this mechanism may be relevant to neutrophil micro-aggregation *in vivo*.

## Introduction

Neutrophils are critical effector cells of the innate immune response and recruited to sites of tissue injury in response to locally generated chemoattractants. Neutrophil migration is a highly regulated process involving complex interactions with the vascular endothelium and tissue stroma. In addition to adhering to the endothelium and their known interactions with platelets, neutrophils also have the capacity to self-associate, a process known as homotypic aggregation (HA) [1]. Physiologically this is believed to contribute to the intravascular pathology associated with disease states such as sepsis [2]. Hence, neutrophil HA can lead to the formation of occlusive plugs within the microcirculation causing tissue hypoxia, hypo-perfusion and endothelial damage [2, 3].

Aminopeptidase N or CD13 is a multifunctional and widely expressed membrane-bound zinc-binding metallopeptidase [4]. CD13 participates in the migration and invasion of cancer and endothelial cells as evidenced in chemotaxis, haptotaxis and extracellular matrix (ECM) invasion assays [5–9]. In these studies overexpression of CD13 has been shown to enhance migration and invasion whilst inhibition of the aminopeptidase impairs these responses. Furthermore, cell invasion into reconstituted basement membrane (BM) with consequent degradation of type IV collagen requires CD13 [5]. Evidence suggests that the enzymatic activity of CD13 is fundamental for promoting cancer and endothelial cell migration and invasion [6, 10]. CD13 also functions as a homotypic adhesion molecule. It mediates monocyte-monocyte and monocyte-endothelial adhesion in response to anti-CD13 monoclonal antibody (mAb) cross-linking on the cell surface [11, 12]. Downstream activation of signal transduction pathways occurs following antibody binding and recent evidence suggests participation by CD13 itself in signalling pathways [11–13].

Human granulocytes express CD13 on their cell surface [14, 15] and we have previously shown the existence of an inverse correlation between neutrophil CD13 activity and the pro-apoptotic efficacy of tumor necrosis factor-α (TNF-α), and the ability of CD13 inhibitors to enhance this response [16]. However, this is the only reported function of CD13 in human neutrophils. This study was designed to evaluate the role of CD13 in regulating migration and HA in human neutrophils. We demonstrate the novel observation of neutrophil HA in response to anti-CD13 mAbs and a resultant reduction in chemoattractant-induced migration through collagen I.

## Materials and Methods

### Isolation of human neutrophils

Neutrophils were isolated from the blood of healthy adult volunteers using dextran sedimentation and discontinuous plasma Percoll^™^ gradients or a 2-step density gradient consisting of Histopaque^®^-1119 and 1077 (Sigma-Aldrich) as described [17, 18].

### Ethics statement

These studies were approved by the Cambridge Research Ethics Committee and the Science, Technology, Engineering and Mathematical Ethical Review Committee of the University of Birmingham. Written informed consent was obtained from all participants and all studies complied with the Declaration of Helsinki.

### Determination of neutrophil CD13 expression

Neutrophils (5 x 10^6^/ml in phosphate buffered saline with cations (PBS+, Sigma-Aldrich)) were stimulated with *N*-formylmethionyl-leucyl-phenylalanine (fMLP, 100 nM; Sigma-Aldrich), interleukin-8 (IL-8), TNF-α (100 ng/ml and 10 ng/ml respectively; R&D Systems) or buffer for 10 or 30 min. Cells were incubated with 100 μl phosphate buffered saline without cations (PBS-, Sigma-Aldrich) containing 5 μl mouse anti-human CD13 fluorescein isothiocyanate (FITC)-conjugated antibody (Clone WM-47; Millipore UK Ltd, FCMAB180F) or an isotype control for 30 min and analysed by flow cytometry (FACSCalibur^™^, BD Biosciences). Mean fluorescence values were calculated from 10,000 events per sample using FLOWJO 7.2.5 software (Tree Star, Inc. Ashland, USA).

### Assessment of CD13 activity

CD13 enzymatic activity was determined as described [16]. Neutrophils (5 x 10^6^/ml in PBS+) were pre-incubated with bestatin (0.1 mM; Sigma-Aldrich) and the monoclonal mouse anti-human CD13 antibodies WM-15 (8 μg/ml; AbD Serotec, MCA1270EL), MY7 (5 μg/ml; Beckman Coulter, 6602626) and WM-47 (1:40 (v/v); Santa Cruz Biotechnology Inc, SC-18891 and Accurate Chemical & Scientific Corp, BYA9643-1) for 30 min. Recombinant human CD13 (rCD13) (200 ng/ml; R&D Systems) was incubated with a concentration range of bestatin (0, 0.001, 0.01, 0.1, 1 mM) for 30 min. Cleavage of the substrate L-Alanine 4-nitroanilide (2 mM; Sigma-Aldrich) was measured over 60 min.

### Neutrophil shape change assay

Neutrophils (5 x 10^6^/ml in PBS+) were pre-incubated with bestatin (0.1 mM), WM-15 (8 μg/ml), WM-47 (1:40 (v/v)) for 30 min prior to stimulation with fMLP or IL-8 for 10 min. Reactions were stopped by the addition of BD CellFIX^™^ (BD Biosciences). The percentage of gated neutrophils that had shifted in FSC-H compared to control unstimulated cells was used to determine the degree of shape change (FACSCalibur^™^). Ten thousand events were collected for each sample and data analysed using FLOWJO 7.2.5 software.

### Neutrophil migration

Neutrophil chemotaxis was assessed using ChemoTx^®^ microplates with a polyvinylpyrrolidone-treated 5 μm pore polycarbonate filter (Neuro Probe^®^, Inc) as described [19]. Neutrophils (5 x 10^6^/ml in Iscove’s modified Dulbecco’s medium (IMDM, Invitrogen) containing 1% (v/v) autologous serum (AS)) were pre-incubated with bestatin (0.1 mM), WM-15 (8 μg/ml), WM-47 (1:40 (v/v)) or buffer for 30 min. Fifty μl of cell suspension was added to each test site on the filter. Migration towards a concentration range of IL-8 (29 μl per well) was quantified after 90 min by counting the number of neutrophils in the lower well using a haemocytometer.

Migration through collagen I (75 μl of 2 mg/ml) was assessed using 6.5 mm Transwell^®^ inserts containing a 5 μm pore polycarbonate filter (Sigma-Aldrich). Gels were prepared according to BD Biosciences ‘Alternate Gelation Procedure for Rat Tail Collagen I’. Neutrophils (6.25 x 10^5^ in 125 μl) were pre-incubated as above for 10 min, in addition to IgG1 (8 μg/ml; R&D Systems, MAB002) and MY7 (5 μg/ml). Migration to IL-8 (650 μl of 100 ng/ml) was quantified in the lower well after 9 hr using a haemocytometer.

Neutrophil migration into collagen I gels was quantified using a phase-contrast microscope with a motorised Z-focus and digital camera using Image-Pro Plus software (Media Cybernetics, UK) as described [20]. All manipulations of gels and microscopy were carried out at 37°C. Collagen I (800 μl in 24 well plates) was equilibrated over 24 hr with an equal volume of IL-8 (100 ng/ml) or Medium-199 (M199, Invitrogen). Fresh M199 was added 30 min prior to neutrophil addition to create a chemoattractant gradient. Neutrophils (1.25 x 10^5^/ml in M199 containing 0.15% (w/v) bovine serum albumin (BSA, Sigma-Aldrich)) were pre-incubated for 10 min with CD13 inhibitors. Digitised Z-stack images were acquired at 3 μm steps, stacking down from the gel surface in four random fields at 30 and 60 min and analysed offline using the same software. The average depth of penetration and the proportion of added cells entering the gel were calculated as described [20].

### Measurement of neutrophil HA

Neutrophils (5 x 10^6^/ml in IMDM containing 1% (v/v) AS) were added to CD13 inhibitors and 100 μl and 200 μl aliquots placed in 96 well flexible plates or 2 ml safe-lock tubes respectively. Fab fragments of WM-15 were prepared using the Pierce^™^ Mouse IgG1 Micro Preparation Kit. Fragmentation was confirmed by SDS-PAGE. Goat anti-mouse (GAM) F(ab)’_2_ fragments (5 μg/ml; Sigma-Aldrich, M0284) were added following a 30 min pre-incubation with WM-47. To assess the effect of kinase and actin polymerisation inhibitors, neutrophils were pre-incubated for 15 or 30 min with LY294002 (10 μM; Cayman Chemical), UO126 (10 μM; Calbiochem) or cytochalasin-D (2 μM; Sigma-Aldrich). Light microscopy assessment of HA was undertaken by capturing well images at the time points indicated using Image-Pro Plus software and quantifying aggregate area using Image J 1.44 software (National Institutes of Health, USA).

Flow cytometry assessment of HA was undertaken after 30 min at 400 rpm (Thermomixer comfort). Reactions were terminated by the addition of BD CellFIX^™^ and gated neutrophils divided into singlets and non-singlets using SSC and FSC area dot plots (LSRFortessa^™^, BD Biosciences). Non-singlets correspond to those particles with high SSC and FSC area [21]. To corroborate the distinction between singlets and non-singlets, the gated neutrophils were also separated by SSC width and SSC height dot plots. Ten thousand events per sample were examined and data analysed using FLOWJO 7.2.5 software.

### rCD13 adhesion assay

A 96 well ELISA microplate was coated with rCD13 (1000 ng/ml) or PBS- (vehicle control) overnight at 4°C. The plate was washed three times and blocked with 1% (w/v) BSA solution for 2 hr at room temperature. Neutrophils (1 x 10^6^/ml in IMDM containing 1% (v/v) AS) were pre-incubated with WM-15 (8 μg/ml) or control for 10 min and 200 μl of cell suspension added to each well in duplicate for 20 min. Plates were washed three times with PBS- and the number of adherent neutrophils was assessed by light microscopy at 100x magnification.

### Statistical analysis

Data represent the mean ± standard error of the mean (SEM) if *n* ≥ 3 or mean ± standard deviation (SD) if *n* = 2. Differences between groups were assessed using repeated measures one-way analysis of variance (ANOVA) with Tukey’s analysis for multiple comparisons or Dunnett’s analysis for comparison with control data. Two-way ANOVA with a Bonferroni post test was used to assess multiple treatments over time. Effects of single treatments were analysed by the Student’s paired and unpaired *t* test. Data were analysed using Prism 5.0 software (GraphPad, San Diego, CA). A *P* value of < 0.05 was considered to be significant.

## Results and Discussion

### Neutrophil expression of CD13

**Fig 1:**
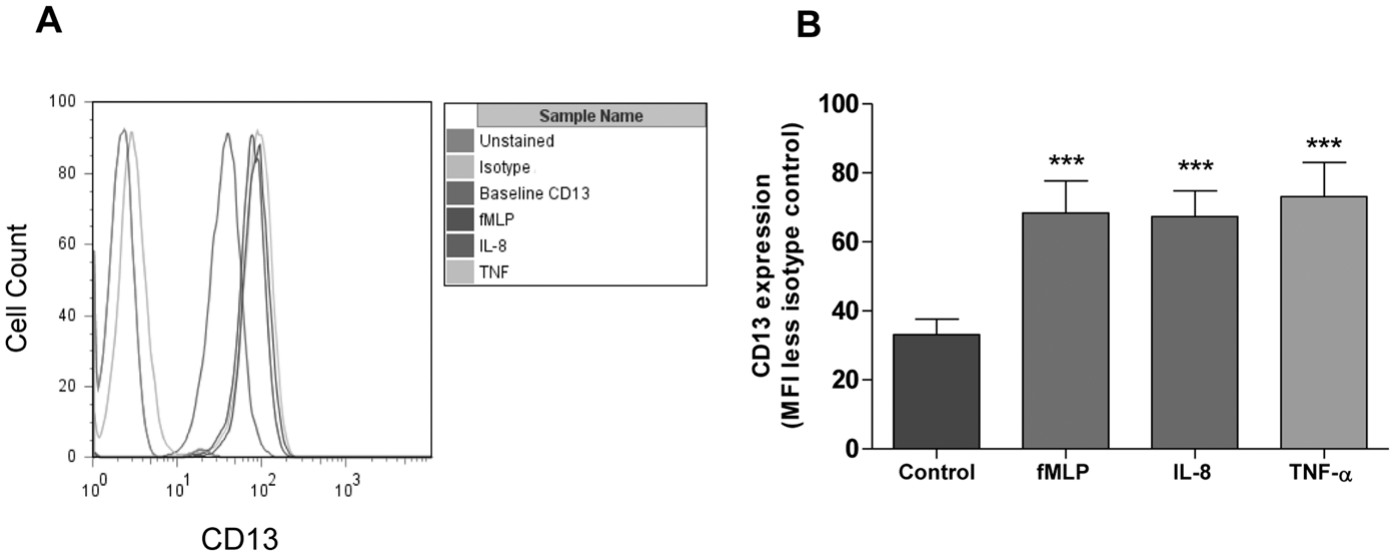
Neutrophil cell surface expression of CD13. Neutrophils were stimulated with fMLP (100 nM), IL-8 (100 ng/ml), TNF-α (10 ng/ml) or vehicle control for 30 min. Representative histograms (A) with quantification of mean fluorescence intensity (MFI) (after subtraction of the isotype control) are shown (B). Data represent the mean ± SEM of *n* = 3 independent experiments each performed in duplicate. ***, *P* < 0.001 vs. control, by repeated measures ANOVA/ Dunnett’s test.

Freshly isolated human neutrophils show clear expression of CD13, as determined by flow cytometry, and the level of expression was increased at 30 min following fMLP, IL-8 or TNF-α stimulation (Fig 1). The increase in expression at the cell surface could be detected as early as 10 min and was approximately twice that of baseline regardless of the agonist used (Fig 1 and S1 Fig). This confirms previously published work demonstrating cell surface expression of CD13 on human granulocyte populations and an enhancement of expression following agonist stimulation [15]. CD13 has been isolated from secretory vesicles of neutrophils, which are mobilised rapidly in response to a wide variety of inflammatory stimuli [22]. The presence of pre-formed CD13 may explain the swift increase in cell surface expression observed in this study.

### Inhibition of CD13 enzymatic activity

**Fig 2:**
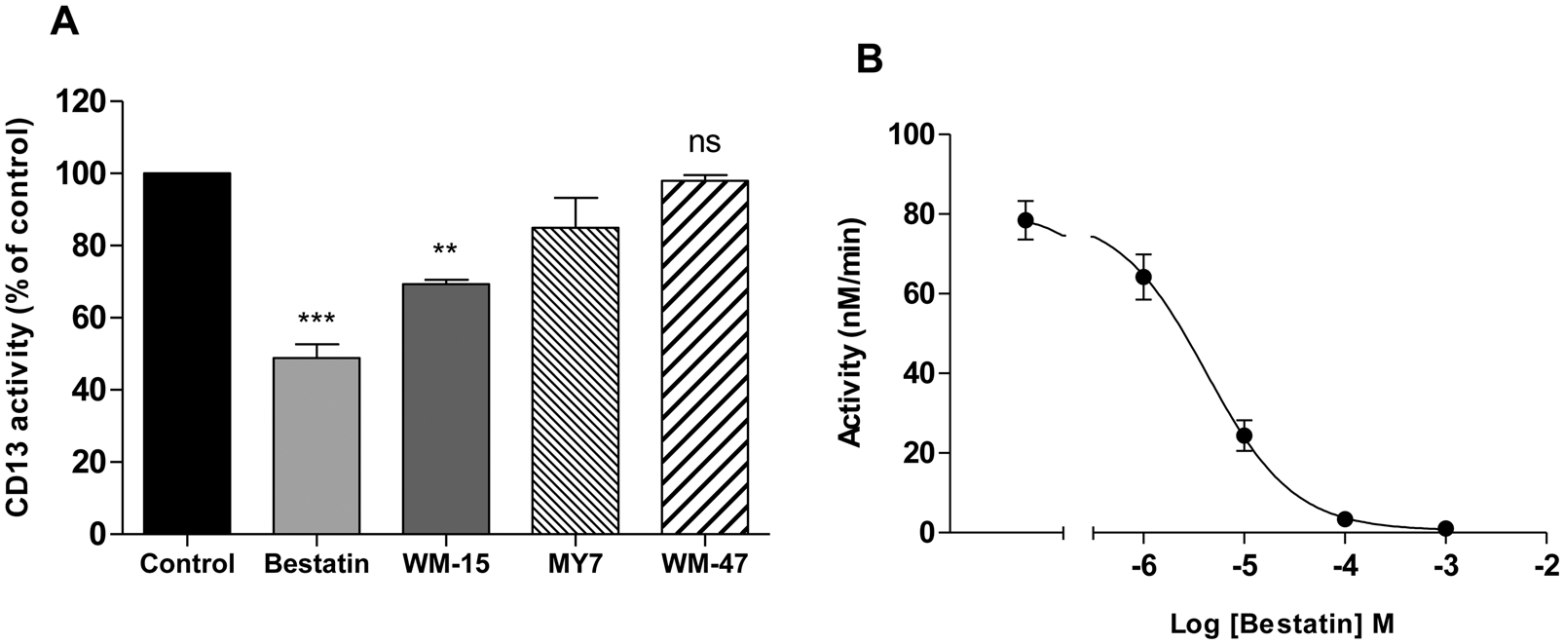
Effect of CD13 ligation on neutrophil and recombinant human CD13 activity. Neutrophils were pre-incubated with bestatin (0.1 mM), anti-CD13 mAb WM-15 (8 μg/ml), anti-CD13 mAb MY7 (5 μg/ml), anti-CD13 mAb WM-47 (1:40 v/v) or vehicle control for 30 min. Cleavage of L-Alanine 4-nitroanilide (2 mM) was measured over 60 min and is expressed as a percentage of the activity observed with control treated cells. Data represent the mean ± SEM of *n* = 7 (Bestatin), *n* = 3 (WM-15 and WM-47) or mean ± SD of *n* = 2 independent experiments (MY7) each performed in triplicate (A). rCD13 (200 ng/ml) was pre-incubated alone or with increasing concentrations of bestatin for 30 min. Cleavage of L-Alanine 4-nitroanilide was measured over 60 min and converted to nmol substrate/min. The mean ± SEM from three independent experiments, each performed in triplicate, are shown. (B). ***, *P* < 0.001, **, *P* < 0.01, ns, non-significant vs. control, by Student’s paired *t* test.

Inhibition of the aminopeptidase activity of CD13 can be achieved by chemical inhibitors or specific antibodies. Bestatin (0.1 mM), a small molecular weight peptide that binds competitively to the active site of CD13, inhibited 51.2% of measured CD13 activity (Fig 2A). This apparent ‘residual CD13 activity’ may be explained by the presence of other (bestatin resistant) cell surface peptidases that are capable of degrading the CD13 substrate and/or the substrate is able to enter the cell and be cleaved by cytosolic peptidases. The demonstration that bestatin inhibits 96.7% of rCD13 activity (at a concentration with equivalent activity to neutrophils in suspension) supports this view and confirms effective inhibition of CD13 (Fig 2B). WM-15, a monoclonal anti-human CD13 antibody, has also been shown to inhibit the enzymatic activity of CD13 on human neutrophils and a comparable degree of inhibition was demonstrated in this study (Fig 2A) [16]. The CD13 mAb MY7 has been reported to partially inhibit CD13 aminopeptidase activity on U-937 cells [11]. In contrast, the CD13 mAb WM-47 has no effect on CD13 enzymatic activity and can be utilised as a selective CD13 control antibody [11]. The degree of enzyme inhibition conferred by MY7 and WM-47 on human neutrophils was 15% and 2.1% respectively, consistent with U-937 cells (Fig 2A) [11]. Epitope mapping studies have demonstrated that WM-15 binds to the zinc-binding domain of CD13 and MY7 binds to a distinct but closely located epitope [23]. WM-47 binds to an epitope separate from the enzymatic active site, explaining the difference in enzymatic inhibitor efficacy of the mAbs [23].

### Role of CD13 in neutrophil polarisation and chemotaxis

**Fig 3:**
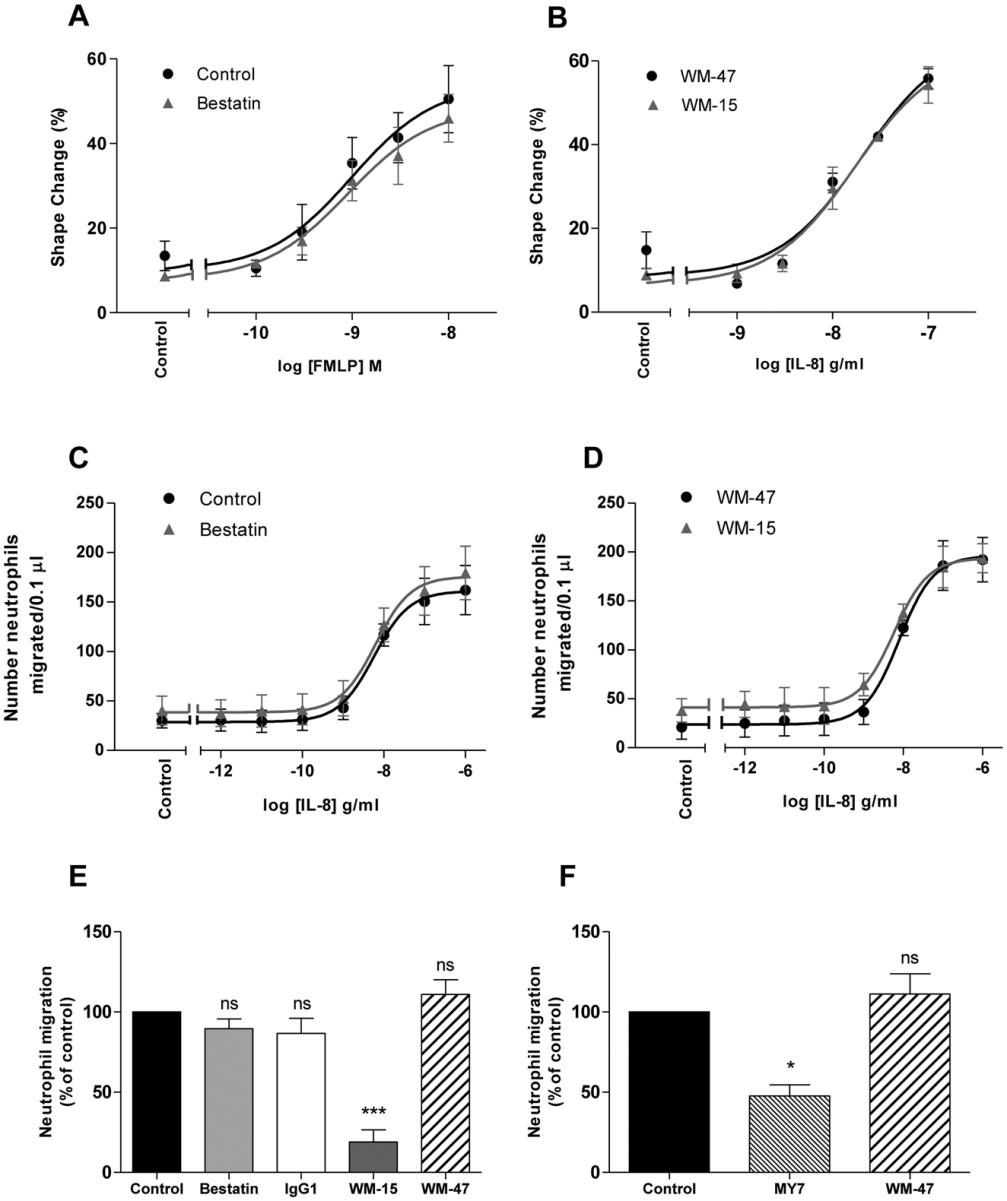
Effect of CD13 ligation on neutrophil shape change and migration. Neutrophils were pre-incubated with bestatin (0.1 mM), WM-15 or IgG1 (8 μg/ml), WM-47 (1:40 v/v), MY7 (5 μg/ml) or vehicle control for 30 min (A, B, C and D) or 10 min (E and F). Cells were stimulated in triplicate with a concentration range of fMLP or IL-8 for 10 min and percentage shape change assessed by flow cytometry (A and B). Chemotaxis was assessed in duplicate using a ChemoTx^®^ plate and migration towards a concentration range of IL-8 quantified after 90 min by manual counting (C and D). Migration through collagen I, gelled in a Transwell^®^ insert, towards IL-8 (100 ng/ml) was quantified in duplicate after 9 hr and is expressed as a percentage of the migration observed with control treated cells (E and F). Data represent the mean ± SEM of *n* = 3 independent experiments (A, C, D, E and F) or mean ± SD of *n* = 2 independent experiments (B). ***, *P* < 0.001, *, *P* < 0.05, ns, non-significant vs. control, by repeated measures ANOVA/Tukey’s test.

Chemoattractants are produced by host cells in response to pathogens or injury and are required for the directed movement of neutrophils to sites of infection or inflammation. Neutrophil shape change or polarisation is a pre-requisite for chemotaxis and can be assessed by non-gradient chemokine challenge [24]. To determine whether CD13 has a role in regulating neutrophil polarisation and chemotaxis, the effect of two inhibitors of CD13 aminopeptidase activity were assessed. Pre-incubating neutrophils with bestatin, WM-15 or the control mAb WM-47, did not modify either the extent or concentration-dependence of chemoattractant-induced neutrophil chemotaxis when measured in a two-dimensional (2D) filter assay, or neutrophil polarisation (Fig 3A, 3B, 3C and 3D). Both of these assays have been validated extensively in neutrophils by our and other groups [19, 25].

The demonstration that CD13 enzymatic inhibition did not impair chemoattractant-induced neutrophil polarisation or chemotaxis contrasts with studies in endothelial and cancer cells. Neutrophil cell surface expression of CD13 is reportedly lower than for certain other cell types and one could speculate that a critical level of CD13 expression is required for 2D migration and that this is not attained in purified human neutrophils [14]. In support of this, Terauchi *et al*. have shown that although bestatin inhibits the migration of CD13 expressing ovarian carcinoma cells, it does not influence the migration of ovarian carcinoma cells with a low level of CD13 expression [9].

### Role of CD13 in neutrophil migration through collagen

Having crossed the venule wall, neutrophils must migrate through the three-dimensional (3D) interstitial matrix en route to the site of inflammation. Collagen I is the predominant collagen type present within the interstitium and hence neutrophil migration through a collagen I gel in the presence of bestatin and WM-15 was assessed using a Transwell^®^ assay. A second CD13 mAb, MY7, which has been shown to have limited CD13 enzymatic inhibitor efficacy was also examined. As shown in Fig 3E and 3F neutrophil migration through a 3D collagen I matrix in response to IL-8 was significantly impaired in the presence of WM-15 and MY7. The chemical enzymatic inhibitor bestatin had no effect suggesting that, in contrast to cancer and endothelial cell invasion, the attenuation in neutrophil migration mediated by WM-15 and MY7 was independent of CD13 enzymatic inhibition. This led us to investigate possible enzymatic independent functions of WM-15 and MY7 on human neutrophils.

### Role of CD13 in neutrophil homotypic aggregation

Aside from its enzymatic activities, CD13 can function as a homotypic adhesion molecule. Using the pro-monocytic cell line U-937, Mina-Osorio and colleagues demonstrated HA in response to the anti-CD13 antibodies 452, WM-15 and MY7 [11].

**Fig 4:**
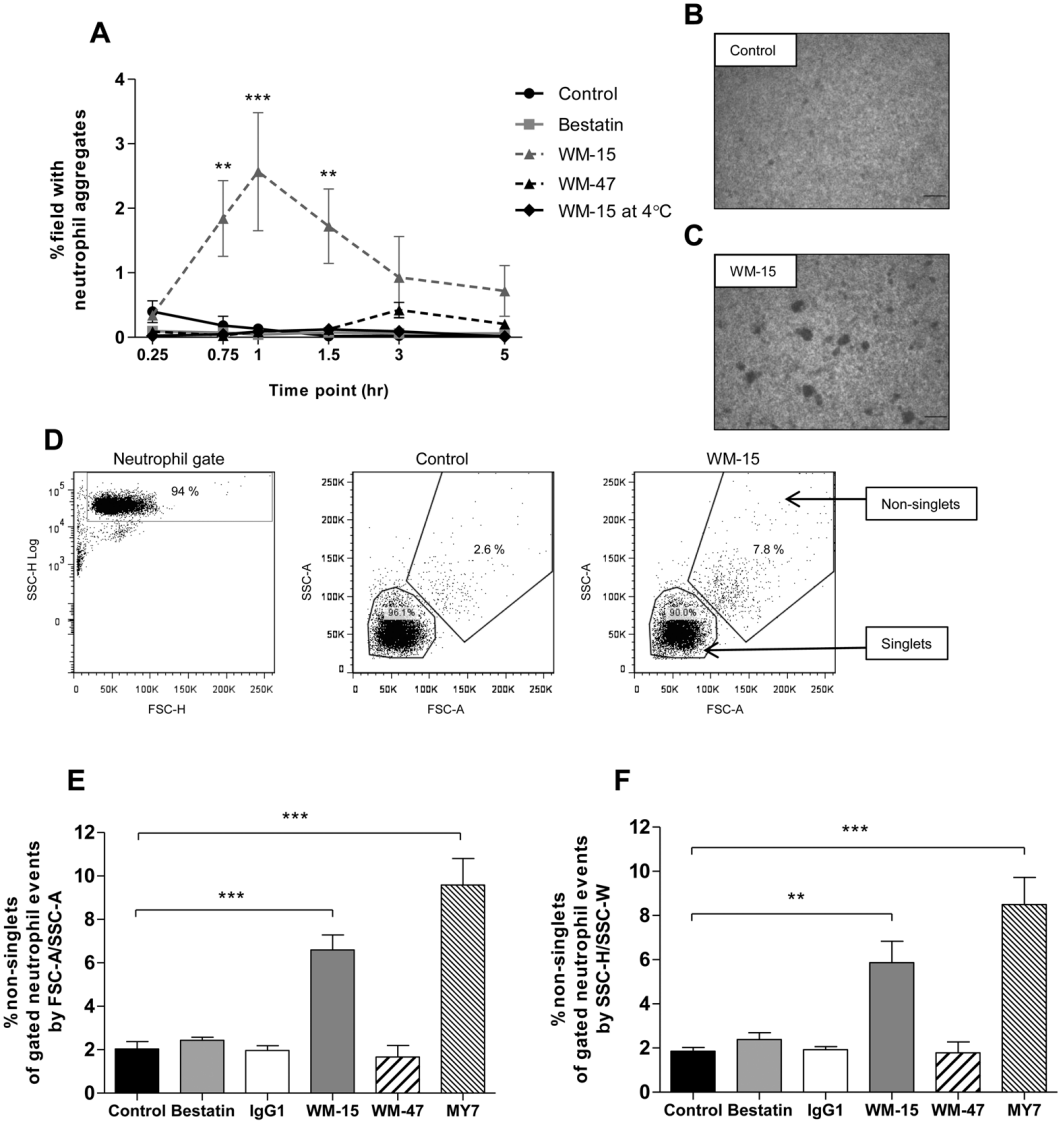
Anti-CD13 mAbs induce neutrophil HA. Neutrophils were incubated with bestatin (0.1 mM), WM-15 or IgG1 (8 μg/ml), WM-47 (1:40 v/v), MY7 (5 μg/ml) or vehicle control. HA was assessed by light microscopy in duplicate at 37°C and 4°C (A) and flow cytometry (E and F) and data represent the mean ± SEM of *n* = 3 independent experiments. ***, *P* < 0.001, **, *P* < 0.01 vs. control, by two-way ANOVA/Bonferroni test (A). Representative photomicrographs after 60 min at 37°C (B and C). Original magnification x 50. Representative SSC area by FSC area dot plots of the fixed neutrophil population separated into singlets and non-singlets (D). Quantification of neutrophil HA at 30 min. ***, *P* < 0.001, **, *P* < 0.01 vs. control, by repeated measures ANOVA/Dunnett’s test (E and F).

To examine the role of CD13 in neutrophil HA a semi-quantitative method using light microscopy and a quantitative method using flow cytometry were employed (Fig 4B, 4C and 4D). As shown in Fig 4A and 4C WM-15 resulted in a significant HA response of purified human neutrophils with aggregation peaking at 60 min. The time course of WM-15-induced neutrophil HA was longer than that observed for chemoattractant-induced HA, which proceeds rapidly and is reversible over a number of minutes [1]. Incubation at 4°C abolished WM-15-induced aggregation (Fig 4A). Neutrophils incubated with WM-15 and MY7 also demonstrated a significant HA response as assessed by flow cytometry (Fig 4E). This effect was recapitulated using SSC width by SSC height flow dot plots (Fig 4F). Distinguishing cell aggregates by SSC/FSC area dot plots of gated cellular events and, for corroboration, SSC width/SSC height dot plots has been established by other investigators [21, 26, 27]. The chemical enzymatic inhibitor bestatin, the CD13 mAb WM-47 and an IgG1 isotype control antibody did not induce neutrophil HA (Fig 4A, 4E and 4F).

The observation that CD13 participates in neutrophil HA has not previously been described. Similar to the pro-monocytic cell line U-937, HA was not observed with the enzymatic inhibitor bestatin or WM-47 [11]. The fact that specific CD13 antibodies induced HA in comparison to the isotype control indicates that this phenomenon is not induced by ‘non-specific’ antibody interactions [28]. Moreover, inhibition of HA at 4°C implies an active, energy-dependant process.

**Fig 5:**
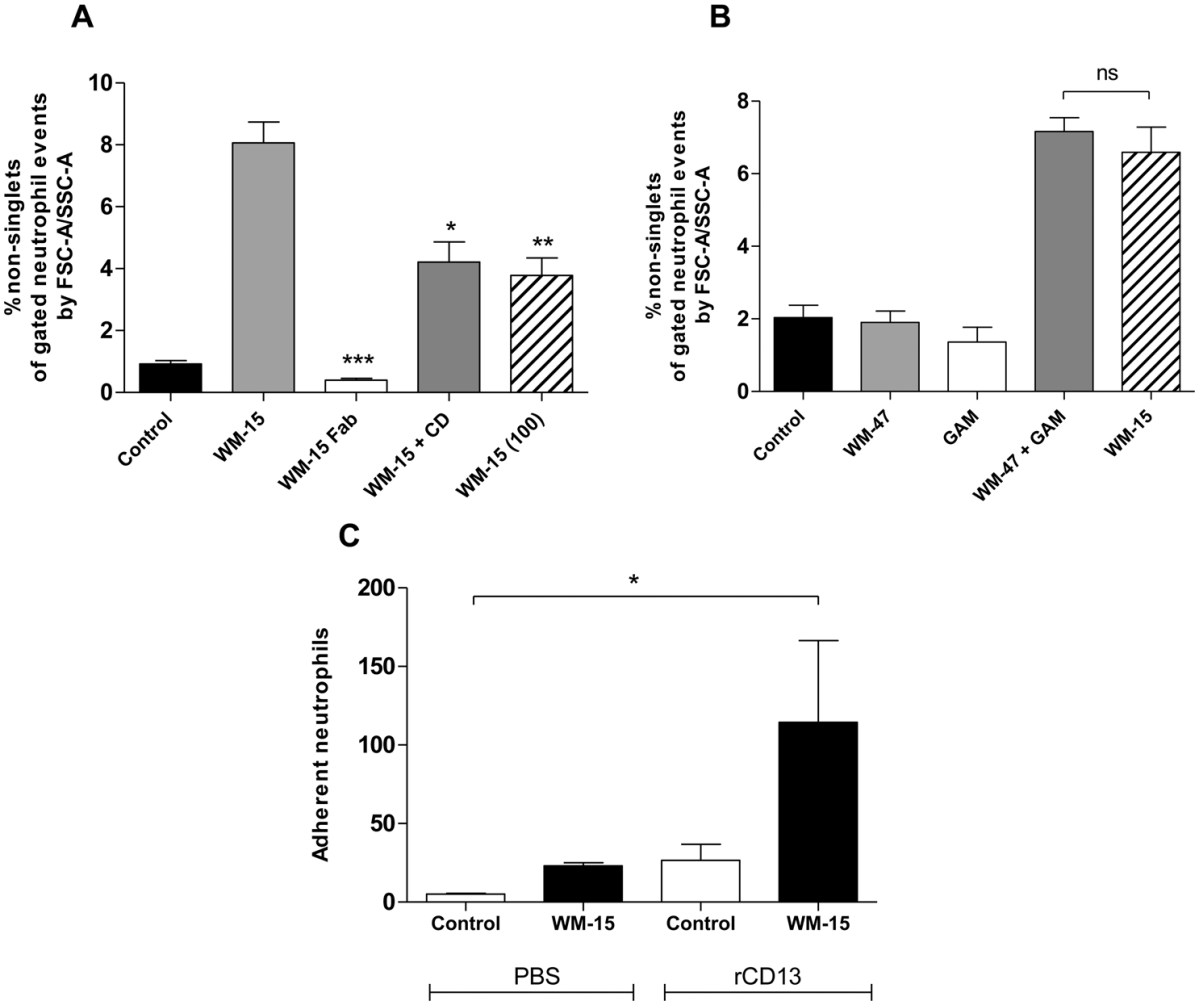
CD13-mediated neutrophil HA depends on CD13 cross-linking, involves actin polymerisation and CD13 directly participates in the adhesive interaction. Neutrophils were pre-incubated for 30 min with cytochalasin-D (2 μM) or vehicle control prior to the addition of WM-15 (8 or 100 μg/ml), Fab fragments of WM-15 (8 μg/ml) or buffer (A). Neutrophils were incubated with WM-15 (8 μg/ml) or with WM-47 alone or followed by a 30 min incubation with F(ab)’_2_ fragments of GAM antibody (B). HA was assessed at 30 min by flow cytometry. Data represent the mean ± SEM of at least *n* = 3 independent experiments (GAM *n* = 2). ***, *P* < 0.001, **, *P* < 0.01, *, *P* < 0.05 vs. WM-15, by Student’s unpaired *t* test (A). ns, non-significant, by Student’s unpaired *t* test (B). Neutrophils were pre-incubated with WM-15 (8 μg/ml) or vehicle control for 10 min and added to PBS- or rCD13 (1000 ng/ml) coated wells in duplicate for 20 min. The number of adherent neutrophils was assessed by light microscopy at 100x magnification. *, *P* < 0.05 vs. control, by repeated measures ANOVA/Dunnett’s test (C).

The ability of certain anti-CD13 antibodies (WM-15 and MY7) but not others (WM-47) to induce aggregation may reflect the recognition of different epitopes, which determines the antibody’s capacity to cross-link CD13 molecules on the cell membrane. This is evidenced from studies by Mina-Osorio and colleagues who observed that whole but not Fab fragments of the antibody 452 induced monocyte-monocyte and monocyte-endothelial adhesion [11, 12]. Consistent with their observations, Fab fragments of WM-15 were incapable of inducing neutrophil HA (Fig 5A). In contrast, cross-linking of the control mAb WM-47 with F(ab)’_2_ fragments of a secondary antibody induced a similar HA response to that of WM-15 alone, confirming that CD13-mediated neutrophil HA is dependent on mAb cross-linking (Fig 5B).

CD13 has been shown to act as a signal transduction molecule [4]. Cross-linking of monocytic CD13 with WM-15 and MY7 evoked a rise in intracellular calcium that was inhibited by tyrosine kinase and phosphoinositide 3-kinases (PI3K) inhibitors [29]. Furthermore, treatment with inhibitors of intracellular kinases, importantly Src family kinases, PI3K and mitogen-activated protein kinases (MAPK), impaired monocyte HA implying a signal transduction-dependent process [11]. Due to its short cytoplasmic domain, it was assumed that CD13 itself was inert and that signalling proceeded through its association with unknown auxiliary proteins or by the regulation of signals initiated by other receptors [4]. However, Subramani *et al*. have recently demonstrated, upon mAb cross-linking, the phosphorylation of CD13 itself and its participation in signalling pathways responsible for increased adhesion [13]. In addition, an association with cytoskeletal adapter proteins was exhibited [13]. Further evidence for direct participation of CD13 in the adhesive process was provided by Mina-Osorio and colleagues who showed that monocyte-endothelial adhesion was impaired by the presence of excess mAb or soluble CD13 [12].

To determine if CD13 itself participates in the adhesive interaction underlying anti-CD13-induced neutrophil HA, aggregation was assessed in the presence of excess WM-15. As shown in Fig 5A, a significant reduction in the HA response was observed with 100 μg/ml WM-15 suggesting inhibition of a homophilic interaction between neutrophil CD13 molecules. The finding that WM-15 treated neutrophils, but not resting neutrophils, specifically adhered to rCD13 further supports a direct CD13-CD13 interaction (Fig 5C). Moreover, WM-15-induced neutrophil HA was significantly reduced following cytochalasin-D pre-treatment, suggesting that microfilament assembly and cytoskeletal alterations have a role in increasing cell adhesiveness (Fig 5A).

**Fig 6:**
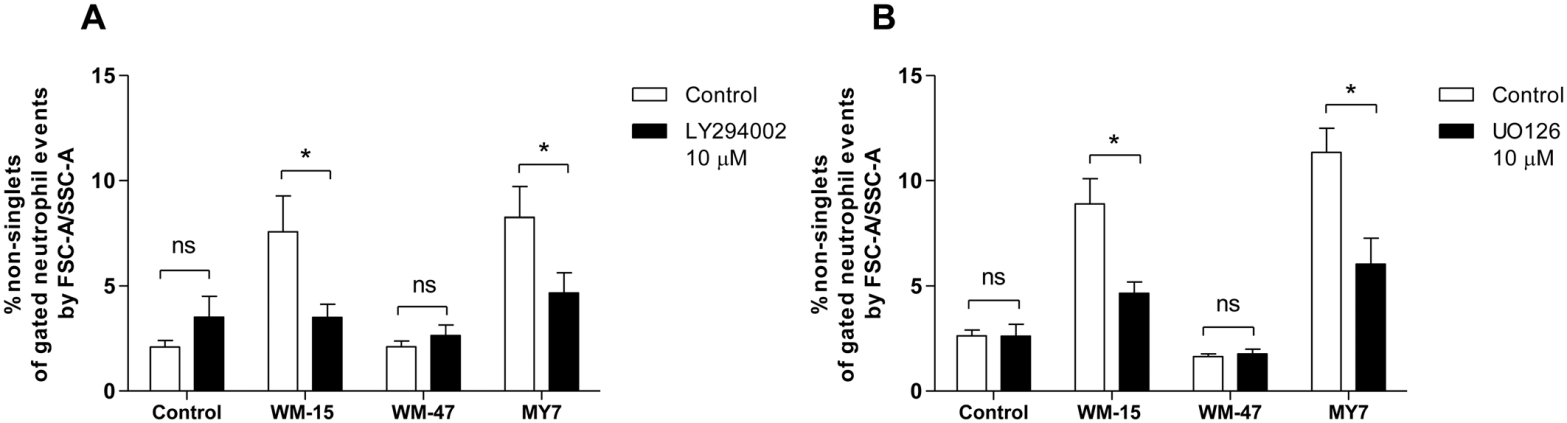
Effect of kinase inhibitors on CD13-mediated neutrophil HA. Neutrophils were pre-incubated for 15 min with LY294002 (10 μM), UO126 (10 μM), or vehicle control prior to the addition of WM-15 (8 μg/ml), WM-47 (1:40 v/v), MY7 (5 μg/ml) or buffer. HA was assessed at 30 min by flow cytometry. Data represent the mean ± SEM of *n* = 4 (A) and *n* = 6 (B) independent experiments. *, *P* < 0.05, ns, non-significant, by Student’s paired *t* test.

To assess the effect of kinase inhibitors on CD13-mediated neutrophil HA, neutrophils were pre-incubated with the PI3K inhibitor LY294002 and the MEK 1/2 inhibitor UO126. MEK 1/2 are the immediate upstream activators of extracellular signal-related kinase (ERK 1/2) [30]. Well-validated concentrations of each inhibitor were taken from previous human neutrophil studies to achieve optimal efficacy and selectivity [30, 31]. LY294002 and UO126 inhibited the HA of human neutrophils induced by WM-15 and MY7, suggesting that both PI3K and ERK 1/2 pathways were involved (Fig 6A and 6B). PI3K and ERK signalling pathways have previously been shown to be involved in chemoattractant-induced and endothelin-1-induced neutrophil HA respectively [32, 33].

### CD13-mediated HA produces a transmigration deficiency across collagen I

**Fig 7:**
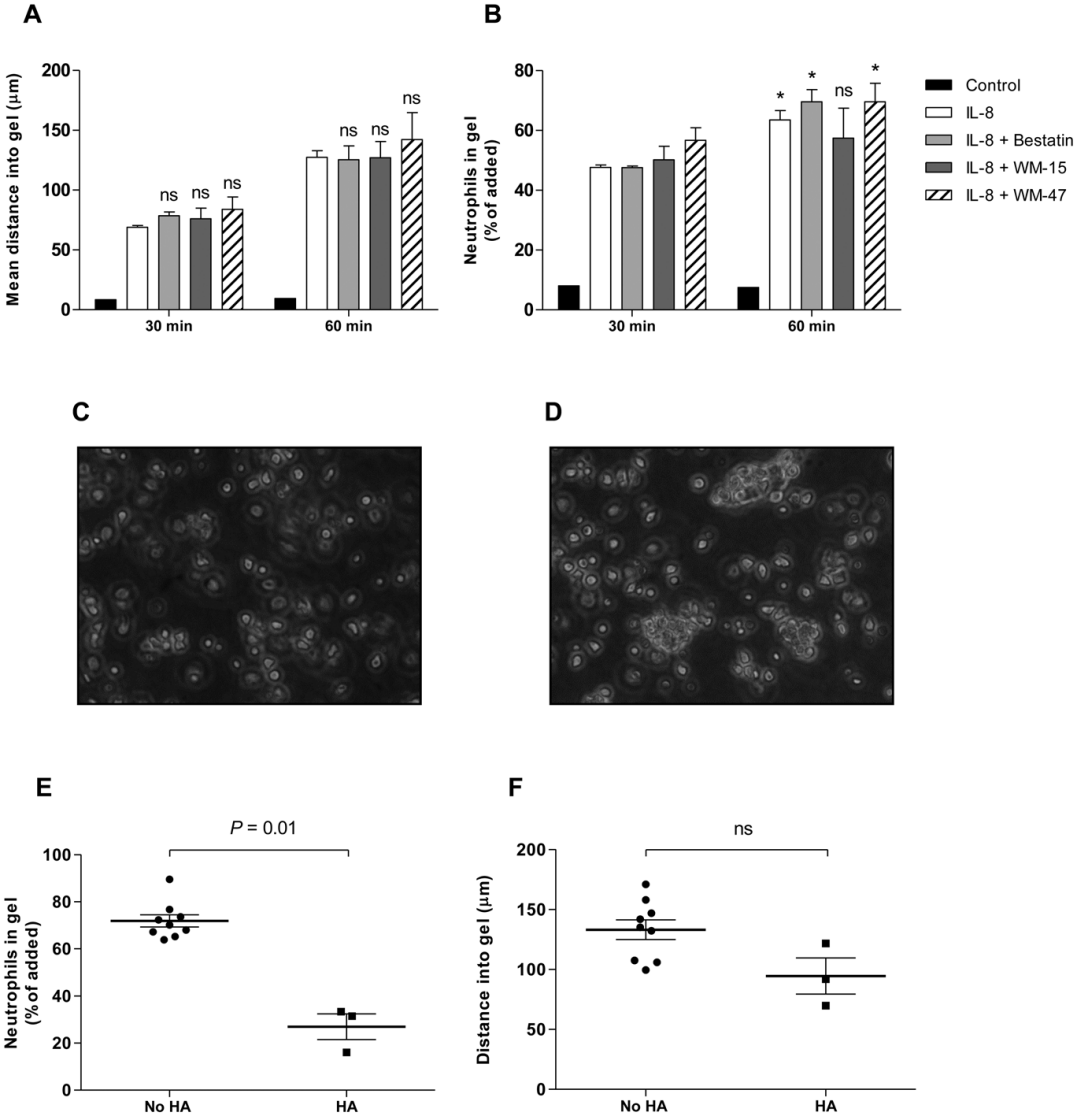
Anti-CD13 mAb WM-15 reduces the proportion of neutrophils entering collagen I. Collagen I gels were equilibrated over 24 hr with IL-8 (100 ng/ml) or media alone (control). Neutrophils were pre-incubated with bestatin (0.1 mM), WM-15 (8 μg/ml), WM-47 (1:40 v/v) or vehicle control for 10 min and the mean distance migrated and percentage cells entering the gel assessed with live cell imaging. Data represent the mean ± SEM of *n* = 3 independent experiments. ns, non-significant vs. IL-8 control, by two-way ANOVA/Bonferroni test (A). *, *P* < 0.05, ns, non-significant vs. 30 min time point, by Student’s paired *t* test (B). Representative gel surface images at 60 min showing WM-15-induced neutrophil HA. Original magnification x 200 (C and D). WM-15 treated neutrophils at 60 min separated according to whether HA was present or not (E and F). ns, non-significant, by Student’s paired *t* test.

To determine whether migration through collagen I was impaired as a consequence of antibody-induced neutrophil HA, direct observations of cell migration were made according to previously published techniques [20]. A collagen I gel treated with IL-8 enabled simultaneous examination of neutrophil migration in response to a gradient of IL-8 together with suitable surface views that allowed the proportion of added neutrophils entering the gel to be determined [20]. Consistent with our chemotaxis data, bestatin and WM-15 had no effect on the mean distance traversed by neutrophils through collagen I over 30 or 60 min (Fig 7A). Neutrophil HA was not detected at 30 min and the proportion of cells entering collagen I was similar across conditions (Fig 7B). At 60 min, however, further entry was inhibited in the WM-15 treated cells and gel surface views at this time showed marked neutrophil HA (Fig 7B and 7D). Where HA was present, the percentage of cells entering collagen I in response to IL-8 was significantly reduced (Fig 7E). Critically, the neutrophils that were able to enter the gel migrated normally (Fig 7F). With other treatments, neutrophil HA was not identified and neutrophil entry into collagen I continued to increase between 30 and 60 min (Fig 7B and 7C).

Similar to our polarisation and chemotaxis data, CD13 enzymatic inhibition did not impair neutrophil migration within collagen I. As outlined above, neutrophil cell surface expression is lower than for certain other cell types, especially tumour cell lines, and a critical level of CD13 expression may be required for 3D migration [14]. Furthermore, fundamental differences between neutrophils and tumour cells exist. Tumour cells migrate predominantly in a protease-dependent mesenchymal manner and the enzymatic activity of CD13 could be anticipated to play a greater role within these cell types [34]. As a consequence of neutrophil HA, we observed that anti-CD13 mAbs (WM-15 and MY7) produced a transmigration deficiency across collagen I by reducing the proportion of cells entering a collagen I gel. The observation that the formation of cellular aggregates impedes migration has previously been demonstrated; hence *Clostridium perfringens* phospholipase C induces heterotypic platelet-neutrophil aggregates, which impair neutrophil movement across an endothelial layer [35].

### Concluding remarks

In summary, these data demonstrate the novel observation of HA of human neutrophils in response to the anti-CD13 antibodies WM-15 and MY7. The HA response is dependent on mAb cross-linking, is signal transduction-dependent, involving PI3K and ERK 1/2, and reduces chemoattractant-induced migration through collagen I matrix.

A consequence of ligand binding is that it can induce dimerisation or clustering of receptors and initiate downstream signalling which may be recapitulated by antibodies [36–38]. Although anti-CD13 mAbs can be used as ligand-mimicking reagents to study CD13-mediated receptor effects, it remains a receptor without a natural ligand [13]. To date, angiotensin III and the Met- and Leu-enkephalins are the only recognised *in vivo* substrates of CD13 and a putative ligand has not been identified [4, 39]. Support for an endogenous ligand was provided by Subramani and colleagues who demonstrated the presence of phosphorylated CD13 in peritoneal lavage cells taken from mice with thioglycolate-induced peritonitis [13]. Moreover, Schmidt *et al*. examined the effect of a specific inflammatory stimulus (*Staphylococcus aureus* supernatant) and demonstrated overexpression of neutrophil CD66b and an increase in HA formation, suggesting that different inflammatory stimuli are capable of inducing different neutrophil phenotypes [28]. By association specific inflammatory reactions may produce unique molecules that are capable of binding to and cross-linking CD13, resulting in CD13-dependent inflammation [40].

Homotypic cellular aggregation is an adhesive phenomenon and, when considering the neutrophil, has both physiological and pathological sequelae. Physiologically neutrophil aggregation may be an important mechanism for the intravascular recruitment of neutrophils at sites of inflammation [41]. Pathologically neutrophil HA can lead to the formation of occlusive plugs within the microcirculation causing tissue hypoxia, hypoperfusion and endothelial cell damage [2, 3]. In severe sepsis neutrophil extravasation and migration into underlying tissue is impaired, possibly as a result of neutrophil aggregation [42]. In contrast to anti-CD13 mAb-induced HA, chemoattractant-induced neutrophil HA proceeds rapidly and is reversible over a number of minutes [1]. This property is anticipated to localise the aggregatory response, minimising the potential detrimental effects of neutrophil HA. Our observation that pro-inflammatory mediators increase cell surface expression of CD13 on neutrophils may suggest that CD13 serves to stabilise and prolong neutrophil HA at sites of inflammation. Impaired transmigration, as demonstrated in this study, would reduce the number of neutrophils able to enter tissues in response to chemoattractants, whilst increasing their intravascular accumulation with consequent risk of vascular occlusion or endothelial damage. As with other aspects of neutrophil function, the balance between beneficial and detrimental effects in inflammation is finely tuned. CD13 may therefore contribute both physiologically and pathologically to pathways already defined for inflammatory cell recruitment.

## Supporting Information

S1 Fig Neutrophil cell surface expression of CD13. Neutrophils were stimulated with fMLP (100 nM), IL-8 (100 ng/ml), TNF-α (10 ng/ml) or vehicle control for 10 min. Quantification of mean fluorescence intensity (MFI) (after subtraction of the isotype control) is shown. Data represent the mean of *n* = 1 experiment performed in duplicate.
(TIF)
