# Supplementary figures and images for: The Aminopeptidase CD13 Induces Homotypic Aggregation in Neutrophils and Impairs Collagen Invasion
Source: PLoS One. 2016 Jul 28;11(7):e0160108. doi: 10.1371/journal.pone.0160108 (PMC4965216; doi:10.1371/journal.pone.0160108)

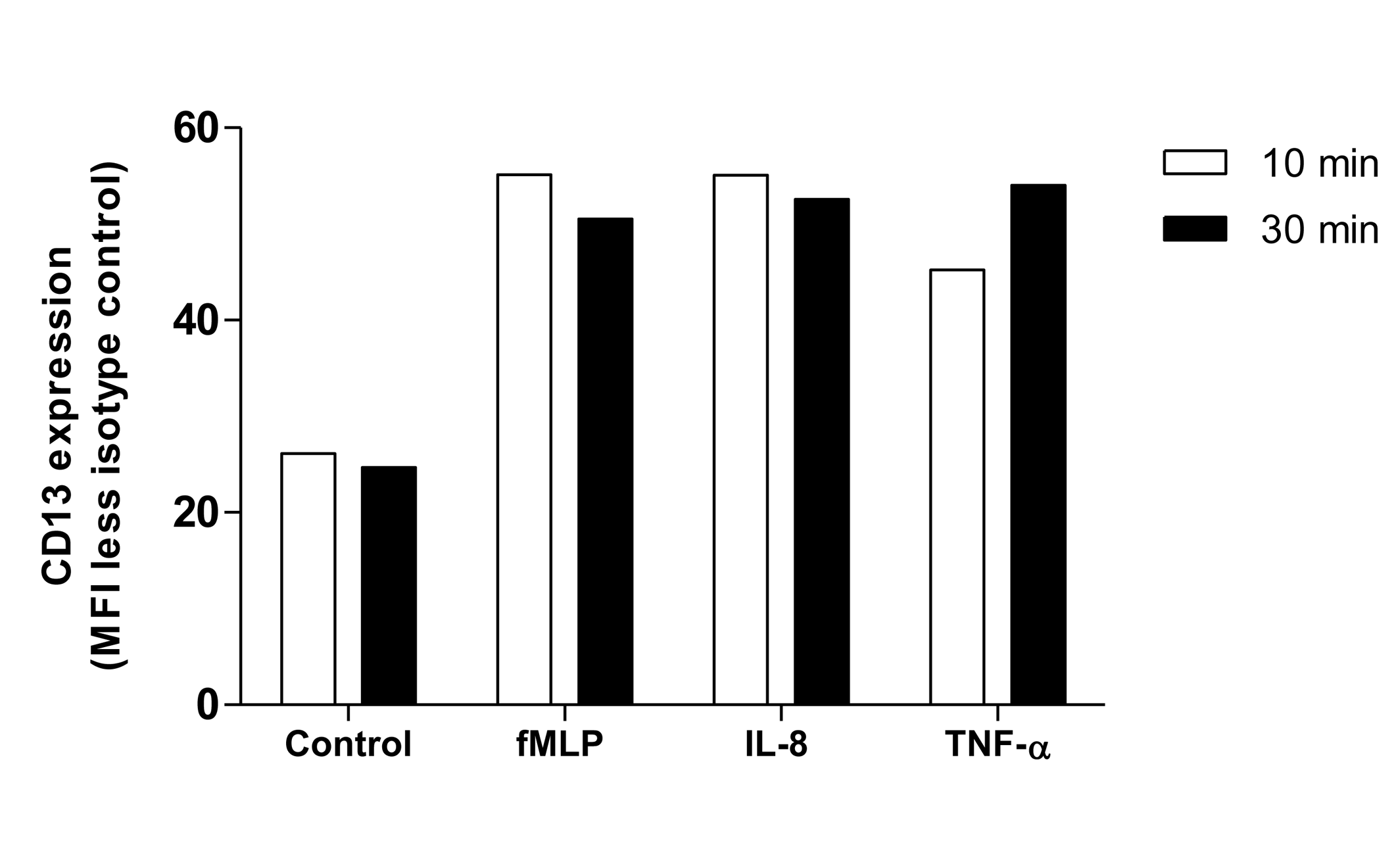

Supplement: S1 Fig — Neutrophils were stimulated with fMLP (100 nM), IL-8 (100 ng/ml), TNF-α (10 ng/ml) or vehicle control for 10 min. Quantification of mean fluorescence intensity (MFI) (after subtraction of the isotype control) is shown. Data represent the mean of n = 1 experiment performed in duplicate. (TIF) [file pone.0160108.s001.tif]
